# Dissecting the function of Atg1 complex in *Dictyostelium* autophagy reveals a connection with the pentose phosphate pathway enzyme transketolase

**DOI:** 10.1098/rsob.150088

**Published:** 2015-08-05

**Authors:** Ana Mesquita, Luis C. Tábara, Oscar Martinez-Costa, Natalia Santos-Rodrigo, Olivier Vincent, Ricardo Escalante

**Affiliations:** Instituto de Investigaciones Biomédicas Alberto Sols, CSIC/UAM, Madrid, Spain

**Keywords:** *Dictyostelium*, autophagy, Atg1 complex, Atg8, Atg101, transketolase

## Abstract

The network of protein–protein interactions of the *Dictyostelium discoideum* autophagy pathway was investigated by yeast two-hybrid screening of the conserved autophagic proteins Atg1 and Atg8. These analyses confirmed expected interactions described in other organisms and also identified novel interactors that highlight the complexity of autophagy regulation. The Atg1 kinase complex, an essential regulator of autophagy, was investigated in detail here. The composition of the Atg1 complex in *D. discoideum* is more similar to mammalian cells than to *Saccharomyces cerevisiae* as, besides Atg13, it contains Atg101, a protein not conserved in this yeast. We found that Atg101 interacts with Atg13 and genetic disruption of these proteins in *Dictyostelium* leads to an early block in autophagy, although the severity of the developmental phenotype and the degree of autophagic block is higher in Atg13-deficient cells. We have also identified a protein containing zinc-finger B-box and FNIP motifs that interacts with Atg101. Disruption of this protein increases autophagic flux, suggesting that it functions as a negative regulator of Atg101. We also describe the interaction of Atg1 kinase with the pentose phosphate pathway enzyme transketolase (TKT). We found changes in the activity of endogenous TKT activity in strains lacking or overexpressing Atg1, suggesting the presence of an unsuspected regulatory pathway between autophagy and the pentose phosphate pathway in *Dictyostelium* that seems to be conserved in mammalian cells.

## 1. Background

Autophagy is an intracellular degradation pathway that occurs in all eukaryotic organisms analysed to date. This process has been associated with numerous pathologies and dysfunctions such as cancer, neurodegenerative diseases, aging, heart and liver diseases, metabolic dysfunction and oxidative stress [1]. There are three types of autophagy: macroautophagy, microautophagy and chaperon-mediated autophagy, of which the first is the best known and will be denoted as autophagy hereafter for simplicity. The autophagy process is initiated with the formation of a cytoplasmic double membrane, the phagophore, which elongates to form a vesicle called an autophagosome. Autophagosomes can engulf a variety of cargos, including portions of the cytoplasm, protein aggregates or damaged organelles, and eventually fuse with lysosomes, where the cargo is degraded. The simple biochemical compounds are transported back into the cytoplasm where they can be used for energy production or recycling. This mechanism is tightly regulated by a number of complexes formed by the so-called autophagy-related proteins (Atg proteins). These complexes regulate the induction, elongation and maturation of the autophagosomes [2]. The first steps of induction and nucleation are controlled by the serine/threonine kinase Atg1/ULK1 and the class III PI3 K VPS34. The activity of the PI3 K generates a PtdIns3P-enriched region at the level of the endoplasmic reticulum (ER) to which the complex formed by Atg2 and Atg18/WIPI is recruited. Concomitantly, Atg12 is covalently bound to Atg5, and the Atg12–Atg5 conjugate form a ternary complex with Atg16L1, which is required for the subsequent lipidation of Atg8/LC3 to phosphatidylethanolamine of the emerging phagophore membrane [2,3].

The first Atg protein described was the serine/threonine kinase Atg1, also known as ULK1 in mammals, UNC-51 kinase in *Caenorhabditis elegans* and CG10967 in *Drosophila melanogaster*. These proteins are highly conserved, especially in the N-terminal kinase domain [4]. Atg1 kinase is directly regulated by target of rapamycin (TOR) via phosphorylation in both mammalian cells and yeast [5]. Atg1, in turn, controls the phosphorylated status of other members of the complex in a species-dependent manner [6]. In yeast, the Atg1 complex is formed by Atg1, Atg13, Atg17, Atg29 and Atg31 [4]. However, in mammalian cells, the ULK1-binding partners required for autophagy include a highly diverged Atg13 and two proteins not present in the yeast model, Atg101 and FIP200. There are no known orthologues of Atg29 and Atg31 in mammalian cells.

Atg101 is a small protein of approximately 200 amino acids that plays a fundamental role in the stability and basal phosphorylation of Atg13 and ULK1 [7,8]. FIP200 (focal adhesion kinase family interacting protein of 200 kDa), also known as RB1CC1 (retinoblastoma 1–inducible coiled-coil 1), interacts with ULK1 and regulates the targeting of Atg16L1 to the autophagosome assembly site [9]. Although there is no FIP200 homologue in yeast, it is generally accepted that Atg17 and FIP200 have equivalent functions.

*Dictyostelium discoideum* is a social amoeba whose developmental cycle takes place in the absence of nutrients, and thus the cells need mechanisms to mobilize resources that enable the maintenance of the cellular homeostasis. As a result, autophagy impairment leads to distinct developmental phenotypes in *Dictyostelium* that can be easily recognized [10]. *Dictyostelium* autophagy shows more similarities to mammalian autophagy than to the one in the yeast model [11]. In *Dictyostelium*, as described in mammalian cells, several autophagosomes can be formed simultaneously in the cytoplasm in close association with the ER. Once formed, they fuse with lysosomes, which are also numerous in contrast with the single huge vacuole of *Saccharomyces cerevisiae* [11]. This similarity extends to the molecular mechanisms as there are a number of autophagic proteins conserved between *Dictyostelium* and mammalian cells that are absent in yeast. For example, Vmp1, an ER-resident protein present in *Dictyostelium* and mammalian cells, plays an essential role in the initial stages of autophagosome formation by regulating the level of PtdIns3P [12–14].

*Dictyostelium discoideum* Atg1 contains an N-terminal kinase domain very similar to its homologues in other organisms, and a C-terminal domain with very limited identity. These two domains are separated by an asparagine-rich segment [15]. Amoebas in which Atg1 has been knocked out are unable to aggregate, and show reduced survival in nitrogen starvation and decreased protein degradation in development, revealing a fundamental role of this protein in autophagy and in the initiation of multicellularity [15,16]. Atg1 is also required for autophagic cell death in a *Dictyostelium* monolayer model [17]. Despite the great potential of *Dictyostelium* in the study of the autophagic machinery, the extent of these studies in this model is still behind what is known in mammalian cells and yeast.

Previous bioinformatics studies revealed the conservation of most Atg proteins in *Dictyostelium* [10]. Specially conserved are those involved in the ubiquitin-like conjugations. Functional studies through gene targeting of Atg8, Atg7, Atg5 and Atg16 clearly support that the ubiquitin-like conjugation process is required for *Dictyostelium* autophagy [15,18–20]. Less clear is the level of conservation of Atg1 complex subunits among eukaryotes as the protein composition and the mechanism of activation differ between species [4]. The analysis of protein similarities in *Dictyostelium* raised reasonable doubts as to whether some of the proposed proteins are the functional counterparts of the yeast or mammalian Atg1 complex subunits. In particular, Atg13 can hardly be recognized by bioinformatics analysis and no clear homologues were obtained for the yeast Atg17 or the mammalian FIP200 proteins [10]. Here, we describe the characterization of the Atg1 complex in *Dictyostelium* through the screening of protein–protein interactions and genetic disruption of the relevant Atg1-interacting proteins. A possible interplay between Atg1 and the pentose phosphate pathway enzyme transketolase (TKT) is proposed.

## 2. Results

### 2.1. Yeast two-hybrid analysis of interactors for Atg8 and related ubiquitin-like conjugation proteins

We have screened a *Dictyostelium* cDNA library in a yeast two-hybrid analysis (Y2H) using Atg8 (DDB_G0286191) as bait. Positive clones were grown and the prey plasmids isolated and sequenced. The interactions were recapitulated in a fresh yeast strain by transforming the corresponding bait and prey plasmids (table 1). A pairwise analysis was also performed between all the putative related ubiquitin-like conjugation proteins and the adaptor protein p62. The selected proteins (Atg3, Atg4, Atg5, Atg7, Atg8, Atg10, Atg12 and p62/SQSTM1) were chosen according to the proposed homologues described previously [10,13] (table 2).

**Table 1.** Yeast two-hybrid screening. Atg 8, Atg1, Atg13, Atg101 and the putative Atg17 and FIP200 were used as baits in yeast two-hybrid screenings using a *D. discoideum* cDNA library. Positive results were obtained for Atg8, Atg1 and Atg101. The minimum region of interaction represents the amino acid sequences encoded in the smallest clone.

bait	interactor	number of clones	minimum region of interaction
Atg8	Atg7 DDB_G0271096	1	301–707
	Atg4 DDB_G0273443	1	471–745
	CrtA, calreticulin DDB_G0283539	2	176–424
	CnrK, cell number regulator DDB_G0292120	2	772–1214
	Dnpep, aspartyl aminopeptidase DDB_G0286149	1	1–484
	DDB_G0289357	10	1–556
	DDB_G0286393	1	1–280
Atg1 (C-terminal)	Tkt DDB_G0272618	5	55–661
	Atg13 DDB_G0269162	6	591–798
Atg101	AreA (DDB_G0279871)	1	1–398

**Table 2.** Pairwise yeast two-hybrid. Pairwise analysis using Atg8 and Atg1 related proteins cloned on pAct and pLex plasmids. Positive interactions are indicated by a plus sign (+). Protein names proposed by previous homology analyses [10].

pAct pLex	Atg3	Atg4	Atg5	Atg7	Atg8	Atg10	Atg12	P62	Atg1	Atg13	Atg101	Atg17	FIP200
Atg3													
Atg4													
Atg5													
Atg8				+				+					
Atg10													
Atg12				+									
Atg16			+										
P62													
Atg1										+			
Atg13											+		
Atg101										+			
Atg17												+	
FIP200													

Interactions among the ubiquitin-like conjugation proteins have been previously described in higher eukaryotes and our results confirmed that most of these interactions also occur in *D. discoideum*. In particular, Atg8 interacts with both Atg7 and the processing protease Atg4. Other expected results obtained in these assays are the interaction between Atg12 and Atg7, which is required for the activation of Atg12, and the interaction between Atg5 and Atg16, which is essential for the localization of the conjugate Atg5–Atg12 to the autophagosome assembly site.

A selective mode of autophagy mediated by selective autophagy receptors (SARs) has been previously described. These receptors are characterized by a short linear sequence motif (LIR motif) responsible for the interaction between SARs and proteins of the Atg8 family. Such a motif can be encountered in the selective autophagy adaptor p62, a protein involved in delivering ubiquitinated protein aggregates to autophagosomes. Therefore, the interaction obtained between this protein and Atg8 in our assay is not surprising. Moreover, p62 was found in ubiquitin protein aggregates containing the marker GFP-Atg8 in *Dictyostelium* Vmp1-deficient cells [13].

Interestingly, we identified five new Atg8 interactors that have not been described previously (table 1). These proteins include the quality control protein Calreticulin, CnrK (a RING zinc-finger domain-containing protein described in *Dictyostelium* as a putative cell number regulator), Dnpep (an aspartyl aminopeptidase that binds two zinc ions per subunit and is highly conserved in human) and the proteins DDB_G0286393 and DDB_G0289357, which show no significant similarity to any other protein from other organisms.

### 2.2. Yeast two-hybrid analysis of interactors for Atg1 complex subunits in *Dictyostelium*

Table 1 shows a summary of the results obtained in the screening of interactors for Atg1 complex subunits using a similar approach as described earlier for Atg8. The proteins selected for the analysis (Atg1, Atg13, Atg101, Atg17 and FIP200) were chosen according to the homology study described previously [10]. Atg1 was cloned in two fragments (N- and C-terminal fragments) due to the presence of DNA repeats in the middle of the gene. Positive results were obtained for Atg1 and Atg101 as described in table 1, while the rest of the baits gave no positive results. A pairwise analysis by yeast two-hybrid was also performed and the results are also summarized in table 2.

Our results show substantial agreement with what was expected based on the described organization of this complex in other organisms [4]. The core of this assembly of proteins is formed by Atg1-Atg13-Atg101, which seems to be conserved. However, we did not detect any interaction for Atg17, FIP200 and the other proteins included in this analysis. In addition to the expected interactions, Atg1 analysis showed an interaction with the protein TKT, an enzyme fundamental in the non-oxidative branch of the pentose phosphate pathway. This interaction was confirmed in five different clones in which the common smallest domain comprised the region between amino acids 55 to 661 of TKT.

Atg101, an autophagic protein conserved in various eukaryotes, but not in *S. cerevisiae*, interacts with a new unknown protein, DDB_G0279871, which does not display homology with any of the previously described Atg proteins but contains a FNIP repeat and a zinc-finger B-box domain. We propose the name AreA (Autophagy regulator A) for this protein. A more detailed analysis of its function in autophagy is described below.

### 2.3. Validation of novel interactions of Atg1 complex proteins by pull-down experiments

**Figure 1.**
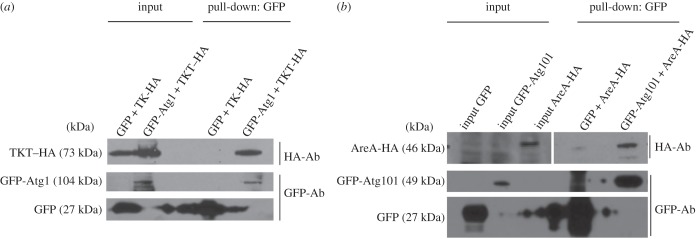
Analysis of new interactions of Atg1 complex subunits by pull-down. (*a*) Protein extracts of *Dictyostelium* cells co-expressing GFP-Atg1 (or GFP as a control) and TKT fused with the HA epitope (TKT–HA) were subjected to pull-down with GFP trap and the interaction analysed by Western blot using anti-HA antibody. The same blot was then used for GFP and GFP-Atg1 detection with anti-GFP antibody. TKT–HA was pulled down by GFP-Atg1 and not by the GFP control. Note: due to the large amount of the GFP control, the signal was expanded to nearby lanes. (*b*) Similar experiment as in (*a*) to detect the interaction between GFP-Atg101 and AreA-HA. In this case, GFP-Atg101 and AreA-HA were expressed in different cells and their respective extracts mixed before performing pull-down experiments.

Once the conservation of the core Atg1 complex subunits in *Dictyostelium* was confirmed, we next wanted to study in more detail the new putative interactors TKT and AreA. To validate the interactions obtained by Y2H, we performed pull-down assays using cell extracts expressing GFP- and HA-tagged proteins (figure 1). GFP-Atg1 and GFP, used as a negative control, were pulled down with GFP-trap and the HA-tagged proteins detected by Western blot. TKT–HA is efficiently pulled down by GFP-Atg1 (figure 1*a*). For the interaction between Atg101 and AreA, we performed a similar approach but in this case we found difficulties to express both tagged proteins simultaneously. Therefore, the different proteins were expressed in separated *Dictyostelium* cells, and the extracts mixed prior to the pull-down assay. GFP-Atg101 pulled down AreA-HA much more efficiently than the control GFP, for which a slight background signal is detected (figure 1*b*).

### 2.4. Subcellular localization of Atg1 complex subunits

**Figure 2.**
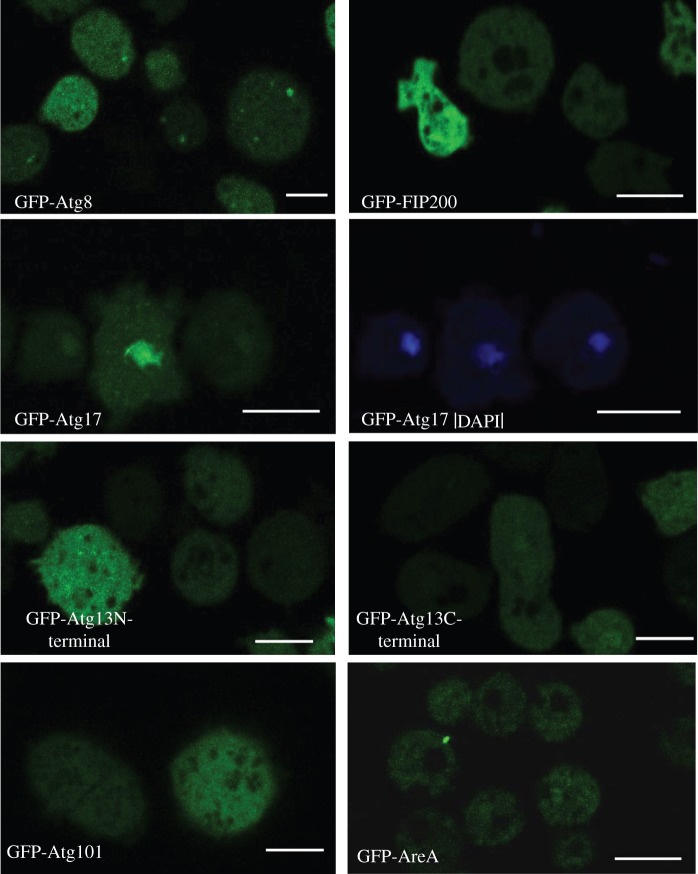
Cellular localization of Atg1 complex proteins by confocal microscopy. For comparison, the autophagic marker GFP-Atg8 is included, which shows the typical autophagosome punctated pattern. Atg101, Atg13-C-terminal, Atg13-N-terminal, FIP200 and Atg17-GFP tagged proteins were expressed and visualized *in vivo* by confocal microscopy. GFP-Atg17 expressing cells were also stained with DAPI for co-localization with nuclei. Scale bars, 10 µm.

We next examined the subcellular localization of the putative autophagy-related proteins Atg13, Atg101, FIP200, Atg17 and AreA to determine if these proteins localize to autophagosomes. For this, the different proteins were tagged with GFP, expressed in wild-type Ax4 cells and analysed by confocal microscopy (figure 2). We found that Atg101, FIP200, Atg13 and AreA are uniformly distributed in the cytoplasm. None of them gave the typical punctuated pattern or labelled any particular structure even when cells were exposed to 30 min of starvation. Although several Atg proteins (such as Atg8 and Atg18) localize to the autophagosomes, giving a typical punctated pattern, others do not show such localization [10]. In particular, Atg1 fused to GFP has been shown previously to have a general cytoplasmic pattern in *Dictyostelium* cells with no specific puncta localization [16]. Therefore, our results show that the rest of the Atg1 complex subunits have a similar non-localized cytoplasmic pattern as observed for the kinase Atg1 in *Dictyostelium*. The only exception to this pattern was found for the putative Atg17 homologue, which preferentially localizes in nuclei, although it also marks the cytoplasm, but with less intensity. This feature is not expected for a protein associated to autophagy as autophagosome vesicles are formed in the cytoplasm.

### 2.5. Gene disruption of the putative Atg1 complex subunits

*Dictyostelium* mutants were generated to investigate the roles of the Atg1 complex proteins in autophagy regulation. Gene disruptions were generated by homologous recombination in Ax4 wild-type strain and confirmed by PCR. The detailed information of this procedure is displayed in electronic supplementary material, figures S1–S5.

**Figure 3.**
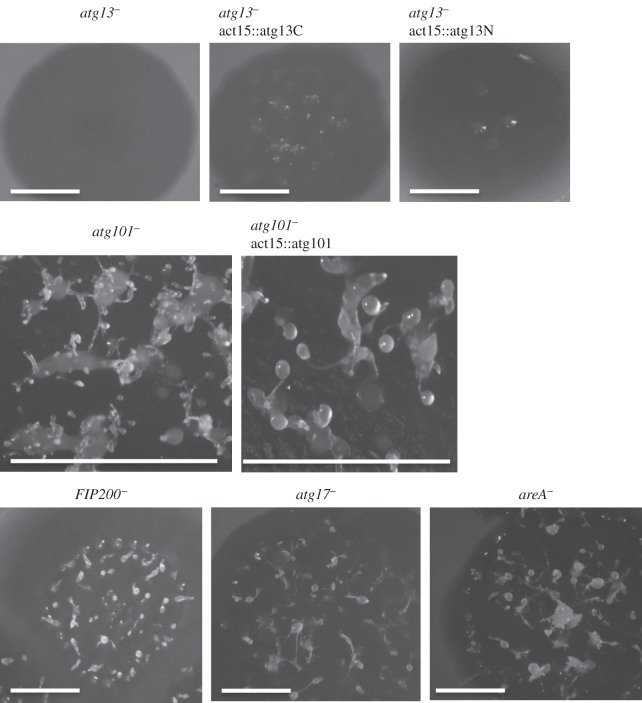
Developmental phenotypes of Atg1-complex mutants. The different strains were deposited on SM plates in association with bacteria. *atg13^−^* mutant showed a lack of aggregation that was only slightly recovered upon expression of the C-terminal region of Atg13. *atg101*^−^ showed the characteristic multi-tip phenotype, with large mounds that develop aberrant multiple small tips on their surface. This phenotype was completely recovered by the re-introduction of Atg101. All other strains showed a normal phenotype. Scale bars, 1 cm.

As the autophagic pathway is required for *Dictyostelium* development [10], we wanted to determine whether the mutant strains were capable of carrying out the full programme of multicellular development. For this, wild-type and mutant cells were plated on SM-agar plates in association with bacteria (figure 3). The resulting phenotypes were analysed 6 days after plating. Among the analysed strains, *atg13^−^* displayed the most accentuated developmental alteration, characterized by the lack of aggregation and the absence of fruiting bodies, similar to what was previously described for the Atg1-deficient strain [15]. This phenotype is in agreement with what was expected given that Atg1 and Atg13 interact and work in close dependence. *atg101^−^* shows a multi-tipped aggregate, which is also indicative of autophagy impairment as demonstrated for other *Dictyostelium* autophagy mutants [15,18,21]. These mutants initiate development and form big mounds that are unable to complete normal fruiting bodies, forming aberrant tips on their surface instead. By contrast, *atg17^−^*, *FIP200^−^* and *areA^−^* strains did not show a developmental phenotype defect. This suggests that either they affect autophagy marginally to a level that is not sufficient to affect development, or, alternatively, they are dispensable for the process. More sensitive and direct assays to monitor autophagy will be used to address this point in the next sections.

### 2.6. Autophagic flux measurements in Atg1 complex mutant strains

**Figure 4.**
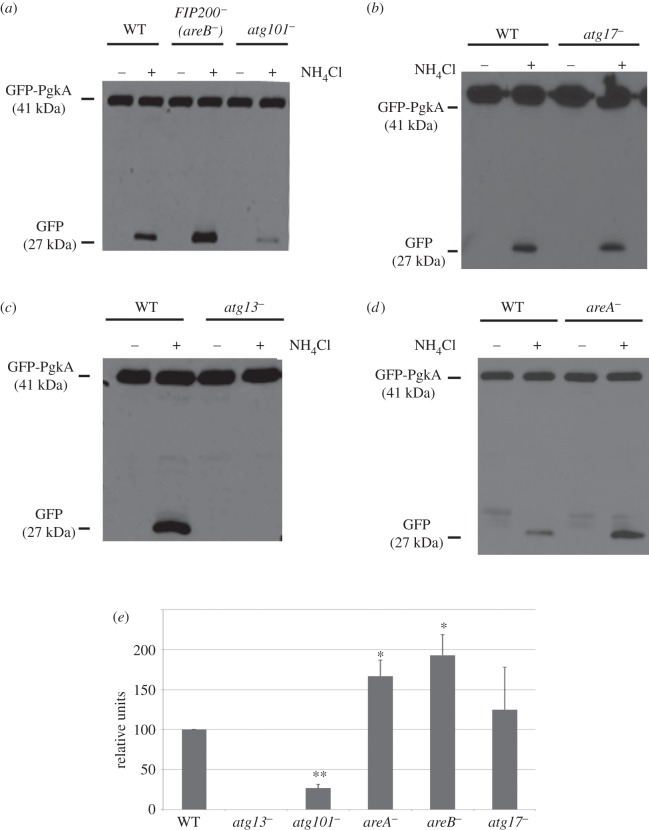
Autophagy flux measurements in the different Atg1 complex mutants. (*a*–*d*) Cells expressing the autophagic substrate GFP-PgkA were incubated in the presence or absence of NH_4_Cl to partially inhibit autophagy. Under these conditions, the level of free-GFP accumulation depends on the autophagy capacity of the cells. A representative experiment for each strain is shown. (*e*) The graph shows the mean values and standard deviations of three independent experiments for each strain. The Western blots were densitometered and the level of free-GFP was normalized with that of the GFP-PgkA and referred to WT. Student's *t*-test shows significant differences (*p < 0.05; **p < 0.01).

The method developed previously in our laboratory to determine autophagic flux in *D. discoideum* is based on the overexpression of phosphoglycerate kinase (PgkA) fused to GFP. This cytosolic protein is taken up passively by autophagy and degraded rapidly in normal conditions. However, if the cells are exposed to ammonium chloride (NH_4_Cl), protein degradation is slowed down due to the increase in lysosomal pH and this allows the detection of cleaved GFP, which is more resistant to degradation than PgkA. The amount of cleaved protein (free-GFP) is assessed by Western blotting analysis and its levels correlates with the autophagic capacity of the cells [22,23]. As shown in figure 4, knocking-out Atg1 complex genes affected autophagic flux to different degrees. The most extreme case was *atg13^−^*, in which autophagy was completely abolished.

The mutant strain *atg101^−^*, as expected by the presence of the multi-tip phenotype, presented a strong decrease in the levels of free-GFP. However, the Atg101 interactor mutant *areA^−^* showed an increased autophagic flux, which suggests an inhibitory effect of AreA on autophagy that could be mediated by the interaction with Atg101. Similarly, disruption of the putative FIP200 gene also resulted in a strongly induced autophagic flux, which also suggests a function as a negative modulator of autophagy. This result is unexpected as FIP200 has been described in mammalian cells as a protein required for autophagy induction [24]. *Dictyostelium* FIP200 protein can be an evolutionary divergent protein that evolved into a different function, or alternatively it may correspond to a different protein not related to mammalian FIP200. The fact that we could not detect interactions between this protein and any of the other Atg1 complex subunits makes the second possibility more likely. Therefore, we propose to name this protein AreB (for Autophagy regulator B).

Atg17 is essential for the activity of Atg1 in yeast [5]. The fact that there is no significant alteration in autophagy flux in a strain lacking the putative *Dictyostelium* Atg17 suggests that this protein is not the functional homologue of Atg17.

### 2.7. Analysis of autophagic markers GFP-Atg8 and GFP-Atg18 in Atg1 complex mutant strains

A more detailed characterization of the autophagic defects was carried out using confocal visualization of autophagic markers. The autophagosome markers GFP-Atg8 and GFP-Atg18 were expressed in wild-type and the different *D. discoideum* Atg1 complex mutant strains. GFP-Atg8 puncta label autophagosomes throughout their initial stages to the degradation stage, as the GFP-Atg8 is attached to inner membrane and degraded in the autolysosomes. By contrast, GFP-Atg18 labels autophagosomes at the initial stages as it is recruited to the site of autophagosome formation as soon as PtdIns3P is generated by the class III PtdIns-3-P kinase VPS34 [25–27].

**Figure 5.**
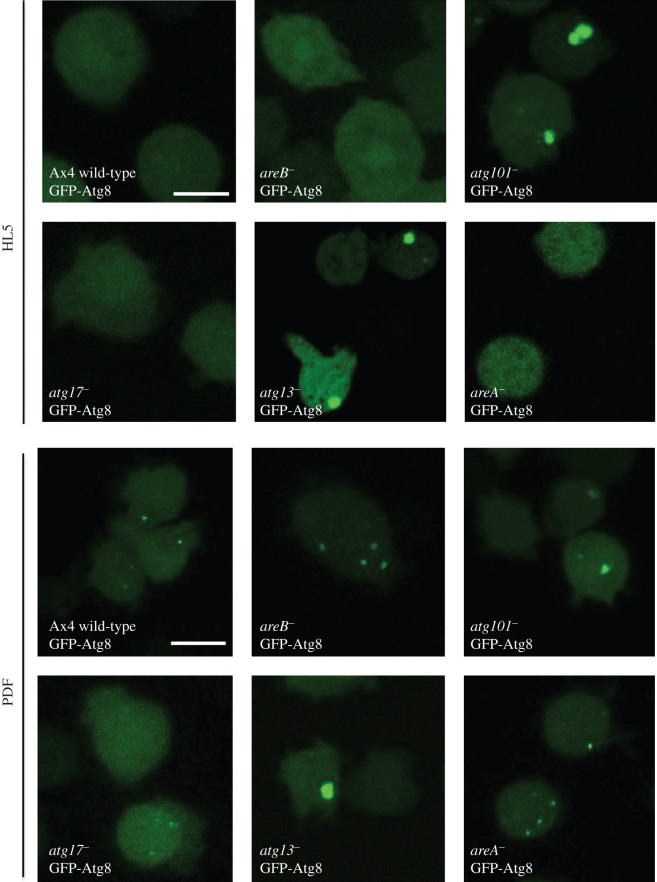
Confocal microscopy analysis of GFP-Atg8. Cells of the indicated strains expressing the marker GFP-Atg8 were subjected to growth (HL5) or starvation conditions (PDF), and the autophagosome pattern was observed under the microscope. *atg13^−^* and *atg101^−^* showed accumulation of aberrant structures irrespective of the growth condition; *areB^−^* displayed large autophagosomes and all other strains showed a normal pattern (see text for details and interpretation). Scale bars, 10 μm.

**Figure 6.**
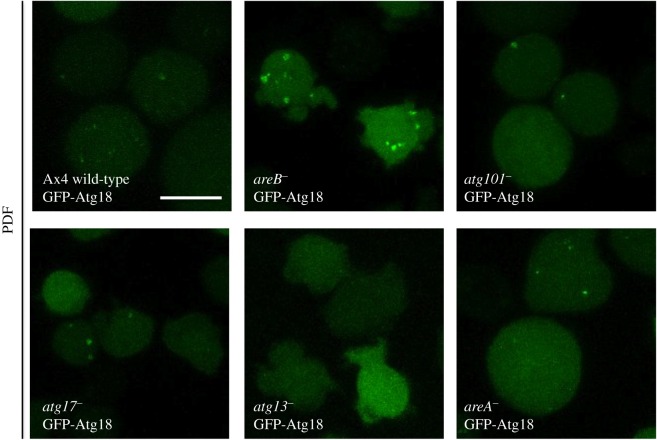
Confocal microscopy analysis of GFP-Atg18. Cells of the indicated strains expressing the marker GFP-Atg18 were subjected to starvation conditions (PDF), and the autophagic pattern was observed under the microscope (no puncta was observed under growth conditions; not shown). *atg13^−^* and *atg101^−^* showed less puncta than WT, and *areB^−^* displayed large autophagosomes. No differences in the pattern were observed for the other strains. Scale bars, 10 μm.

Figures 5 and 6 illustrate representative experiments under growth (HL5) and starvation conditions (PDF). *atg13^−^* mutants showed the most altered pattern with the formation of large aberrant aggregates of GFP-Atg8 and the absence of GFP-Atg18 puncta. The aberrant pattern of GFP-Atg8 has been described before in other *Dictyostelium* autophagic mutants [13,15,19]. This indicates a strong impairment of autophagy that corroborates the results of the flux assay. *atg101^−^* mutant strain also showed some abnormal aggregates of GFP-Atg8 although not as large as in *atg13^−^.* In contrast, the disrupted strain for AreB (previously named as FIP200) showed unusually large autophagosomes with a clear vesicle-like structure (figure 6). This result is in agreement with the increased autophagic flux observed in the proteolytic cleavage assay and suggests that *areB^−^* mutant has increased autophagic capacity. In the strains *atg17^−^* and *areA^−^*, we did not detect clear differences in the patterns.

### 2.8. Effects of Atg1 on transketolase activity

TKT, a thiamine diphosphate-dependent enzyme is the key rate-limiting enzyme of the non-oxidative branch of the pentose phosphate pathway [28]. The fact that this enzyme interacts with Atg1 opens a whole new set of questions. Does TKT have an influence in autophagy? Is Atg1 regulating TKT activity?

**Figure 7.**
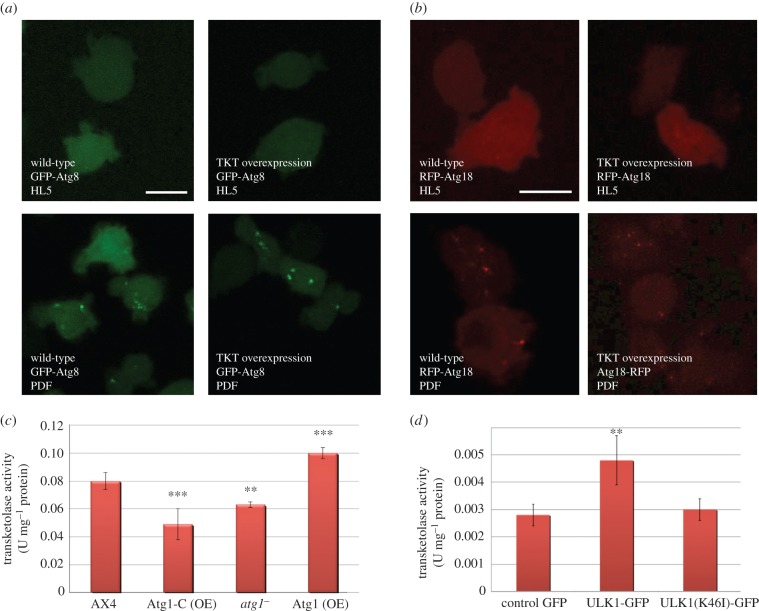
Atg1 and TKT activity. Effect of TKT overexpression in the pattern of (*a*) GFP-Atg8 and (*b*) RFP-Atg18 in *Dictyostelium* cells. Effect of Atg1 on TKT activity in *Dictyostelium* and mammalian cells HEK293T. (*c*) *Dictyostelium* cells deficient in Atg1 (*atg1*^−^), over-expressing Atg1 (Atg1 (OE)) and overexpressing the C-terminal region of Atg1 (Atg1-C (OE)) were lysed, and the activity of the endogenous TKT was measured. (*d*) TKT activity in human HEK293T cells transfected with expression plasmids for ULK1-GFP or the kinase dead form ULK1(K46I)-GFP. Results represent the means and standard deviations of at least three independent experiments. Student's *t*-test shows significant differences (*p < 0.05; **p < 0.01; ***p < 0.001). Scale bars, 10 μm.

To verify the first possibility, the pattern of autophagy markers GFP-Atg8 and RFP-Atg18 was analysed by confocal microscopy in wild-type and TKT overexpressing cells (figure 7*a*,*b*). The overexpression of TKT did not induce GFP-Atg8 or GFP-Atg18 puncta in HL5 growth media. In addition, the overexpression of TKT did not alter the puncta pattern under starvation (PDF) compared with wild-type, which suggests that TKT does not regulate autophagy neither in basal nor in starvation conditions.

In order to study the other possible functional connection between Atg1 and TKT, we measured the activity of TKT in conditions of altered Atg1 activity (figure 7*c*). Our result indicates that genetic alterations in *Dictyostelium* *atg1* have an effect on TKT activity. There is an increase in activity in cells overexpressing Atg1. By contrast, TKT activity decreases in cells in which *atg1* has been deleted or expresses an Atg1-C-terminal construct that lacks the kinase domain. An Atg1 kinase mutant has been shown previously to act as a dominant negative in *Dictyostelium* [16]. No significant differences of glucose-6-phosphate dehydrogenase (G6PDH) activity were detected in any of these strains (data not shown).

Next, we wanted to determine if the modulation of TKT activity by Atg1 has been conserved in human cells. To address this question, we measured the activity of TKT in human embryonic kidney (HEK293T) cells in which ULK1 was overexpressed. The results displayed in figure 7*d* lead us to conclude that human ULK1 positively regulates TKT and that this regulation depends on Ulk1 kinase activity as the expression of a dead kinase mutant does not alter TKT activity.

## 3. Discussion

### 3.1. Analysis of Atg8 interactors reveals a high conservation of the autophagy network and putative new regulators

**Figure 8.**
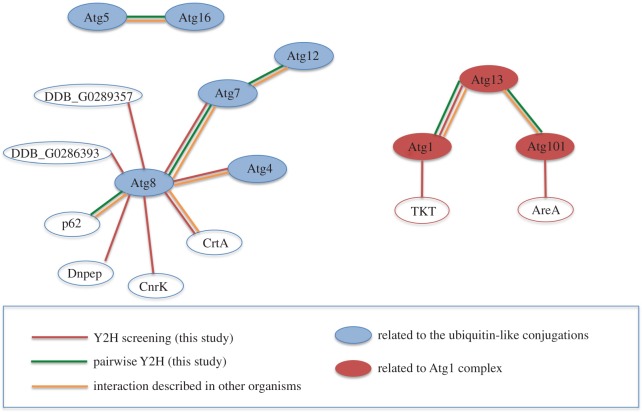
Summary of interactions obtained by Y2H analysis. The data obtained from Y2H screenings and Y2H pairwise analysis are summarized in the diagram, representing the interactions between the proteins as connected coloured lines. The colour code also informs of interactions described in other organisms.

Atg8 is a ubiquitin-like protein that is a fundamental component in the autophagy machinery. The function of this protein depends on its association with several other proteins in a cascade of ubiquitin-like reactions that eventually conjugate Atg8 to the autophagosome membrane. Our analyses have identified fundamental interactions among these components that support the conservation of this process in *Dictyostelium*. Figure 8 summarizes all the interactions obtained.

Apart from these expected Atg8 interacting proteins, we also encountered new potential interactors. Calreticulin is a molecular chaperone in the ER that also has a function in calcium homeostasis. Although this protein is ER located, it has been detected in other cellular locations such as mitochondria, nucleus and the cytosol, and associated to new roles in processes like apoptosis or inflammation [29]. It might also be possible that the interaction takes place at the omegasome, as a physical connection has been described to occur between the phagophore membrane and the ER [30]. Interestingly, there is a mammalian LC3 homologue, known as GABARAP, that has been identified as an interactor of calreticulin in mammalian cells [31], the functional significance of this interaction being still unknown. The conservation of the interaction between calreticulin and Atg8/LC3 in *Dictyostelium* and mammalian cells supports the hypothesis that this protein might act as an autophagy regulator, an issue that will need further investigation.

Aspartyl aminopeptidase, DNPEP, catalyses the removal of an N-terminus aspartate or glutamate of protein substrates [32,33], a process necessary for intracellular peptide metabolism that has been implicated in blood pressure regulation.

CnrK (cell number regulator) is a protein originally described as a suppressor of SmlA, a protein of unknown function whose disruption generates *Dictyostelium* developmental structures of small size [34]. CnrK has protein similarity (*E*-value 3 × 10^−20^) to several E3 ubiquitin protein ligases containing the typical RING zinc-finger domain and the IBR domain. Interestingly, the first hit when compared with the human proteome is ARIH1 (also known as HHARI, human homologue of Ariadne). This E3-ligase has recently been proposed to serve redundant roles with Parkin, another E3-ligase that has been shown to play an essential role in mitophagy (the specific degradation of mitochondria by autophagy) [35].

The *Dictyostelium* proteins DDB_G0289357 and DDB_G0286393 show no clear homology to any protein from yeast or mammalian cells, although the presence of LIR motifs and the interaction with Atg8 suggest that these two proteins might have a role in autophagy.

### 3.2. The core Atg1 complex is conserved in the *Dictyostelium* model

The protein composition of the Atg1 complex has been conserved in different species, as shown in table 3. However, there are some subunits that appear to have diverged during evolution. The common core subunits of the complex are formed by the kinase Atg1, which shows high amino acid conservation in all analysed species, including *Dictyostelium* [15], and the Atg13 protein. Atg13, however, has shown a very low similarity at the amino acid level in the different species, which makes the identification of the homologues a difficult task. As a matter of fact, the *Dictyostelium* Atg13 protein does not show an overall significant similarity to the yeast or mammalian Atg13 but was annotated in Dictybase as having a short sequence of similarity to Atg13. This led us to include this protein in our analysis. The interaction analysis and the functional studies presented here indicate that the putative *Dictyostelium* Atg13 is a bona fide homologue. Atg13 function has been always linked to a direct regulation of Atg1 kinase, thus it is expected that the disruption of the gene results in similar defects to the ones observed in the Atg1 mutant. Our results are consistent with this idea as the observed phenotypes, the lack of aggregation and the absence of detectable autophagy are similar to those observed in Atg1 mutant [15]. This contrasts with the most common multi-tip phenotype observed in other autophagic mutants [10].

**Table 3.** Summary of the proposed *Dictyostelium* Atg1 protein complex. The previously proposed FIP200 and Atg17 homologues were not included as our studies suggest that they are not the functional homologues (see the Results and Discussion sections).

	*S. cerevisiae*	*H. sapiens*	*D. discoideum*
Atg1	Atg1	ULK1/2	Atg1 (DDB_G0292390)
Atg13	Atg13	Atg13	Atg13 (DDB_G0269162)
Atg17	Atg17	—	—
Atg29	Atg29	—	—
Atg31	Atg31	—	—
Atg101	—	Atg101	Atg101 (DDB_G0288287)
FIP200		FIP200/RB1CC1	—
other regulators			AreA (DDB_G0279871) AreB (DDB_G0268498)

Atg101 is a small protein discovered in mammalian cells that seems to be absent in yeast [7,8]. Atg101 interacts with ULK1 in an Atg13-dependent manner, stabilizing the expression of Atg13 in the cell and protecting it from proteasomal degradation [8]. Disruption of the homologous protein in *Dictyostelium* leads to the characteristic multi-tip phenotype, which suggests an autophagy blockage but not to the extent observed in *atg13^−^* or *atg1*^−^. The autophagy machinery appears to be functional to a lesser extent, as confirmed by the autophagic flux measurements, where autophagy is decreased to approximately 20% in comparison with wild-type. In summary, the analysis of protein interactions between the putative Atg1 complex subunits suggests that the core complex is formed by the proteins Atg1, Atg13 and Atg101 in *D. discoideum*.

### 3.3. DDB_G0295673 is not the functional Atg17 homologue

Atg17 was described in yeast as a modulator of the size of autophagosomes as Atg17 mutants showed fewer and smaller autophagosomes [36]. We did not find any interacting protein for the putative *Dictyostelium* Atg17, and the mutant strain showed a wild-type phenotype and no significant alteration in autophagy flux. Given all this, we believe that the putative Atg17 described in this work is not the functional homologue of the yeast Atg17.

### 3.4. The putative *Dictyostelium* FIP200 (AreB) functions as a negative regulator of autophagy

Different studies of FIP200 in various types of mammalian cells showed the pivotal role of this protein in autophagy and other cellular mechanisms. Starvation-induced autophagy is almost completely lost in mouse embryonic fibroblasts lacking FIP200. These cells also showed the accumulation of the protein p62, which added to the previous results indicate that autophagy is profoundly impaired [37]. Additional work using haematopoietic cells lacking FIP200 showed a rise in the number of mitochondria and p62, and also an increase in reactive oxygen species [38].

These observations are not consistent with the ones we have observed for the putative FIP200 (now named as AreB) from *D. discoideum*. The disruption of the *areB* gene leads to an increased autophagic capacity, as demonstrated by the increase in the autophagic flux and the size and number of puncta observed by the use of GFP-Atg8 and GFP-Atg18 markers. The low similarity at the level of amino acid sequence between *Dictyostelium* AreB and mammalian FIP200 makes it conceivable that this protein has diverged during evolution and might play a different role in *Dictyostelium* autophagy.

### 3.5. The zinc-finger B-box and FNIP containing protein DDB_G0279871 (AreA) interacts with Atg101 and regulates autophagy

We propose the name AreA (Autophagy regulator A) for the protein corresponding to DDB_G0279871. AreA appears to be a new Atg1 complex protein that has not yet been described in other model organisms. *areA*^−^ mutant strain is phenotypically similar to the wild-type and the observation of autophagosome markers GFP-Atg8 and GFP-Atg18 did not reveal obvious alterations in autophagosome shape and size. Despite these similarities to wild-type, we can observe that the autophagy flux in these cells is increased, which may be indicative of a regulatory but not essential role of this protein in the complex.

Analysis of this protein using BLAST did not recognize clear homologues in mammalian cells or yeast. However, it contains three conserved domains, a B-box zinc-finger domain, a FNIP domain and a domain of unknown function called DUF4559. The first two domains are believed to mediate protein–protein interaction in proteins of diverse functions and thus are not informative about a possible biochemical function. The DUF4559 motif is present in the human protein of unknown function CXorf38. We also analysed possible similarities at the structural level using HHpred (homology detection and structure prediction at http://toolkit.tuebingen.mpg.de/hhpred). The first hits are proteins of the TRIM family of ubiquitin E3 ligases and the homology is restricted to the B-box domain. Interestingly, the pattern of developmental expression of this gene as described in dictyExpress (https://dictyexpress.research.bcm.edu/landing/) shows high levels of expression at vegetative stage, which strongly and rapidly decrease over the next 4 h of starvation, a time period in which autophagy is activated. Further studies will be necessary to determine if AreA regulates the availability or stability of Atg101 during the first hours of development.

### 3.6. Transketolase and autophagy

Atg1 kinase protein interacts with the pentose phosphate pathway enzyme TKT. Due to the importance of these two proteins in their respective routes, we decided to investigate this relation in more detail. Imaging studies using confocal microscopy suggested that TKT has no effect on autophagy, excluding this enzyme as a direct modulator of this pathway in *Dictyostelium*. However, our results indicate that alterations in Atg1 levels affect TKT activity in a way that suggests a direct regulation, although we have not been able to prove a possible phosphorylation of TKT by the kinase Atg1. Further investigations will be necessary to address if this regulation is direct.

Besides the importance of TKT in the metabolic flux, only very recently has a post-translational regulation of TKT activity in mammalian cells been described [39]. Amino acids, via the mammalian target of rapamycin (TOR complex) TORC2, regulate Akt activity and Akt association and phosphorylation of TKT, increasing its activity to provide high de novo purine synthesis required for active growth. As Atg1 is negatively regulated by the other TOR complex TORC1 in mammalian cells, this might be another related pathway to regulate TKT, and therefore to coordinate amino acid availability with glucose utilization, purine synthesis, and RNA and DNA synthesis. In *Dictyostelium*, both TORC1 and TORC2 complexes have been described [40], but whether the canonical TORC1/Atg1 pathway functions in *Dictyostelium* in the same way as described in other systems is not known.

Starvation triggers *Dictyostelium* development, which takes place in the absence of cell growth. However, developing cells are metabolically very active, as they need to accomplish highly energy-demanding tasks, such as cell chemotaxis during aggregation and cell differentiation. It is possible that the pentose phosphate pathway provides NADPH and nucleotides required for these processes. In fact, it has been described that an active pentose phosphate pathway exists during *Dictyostelium* development [41]. It has also been described that there is an increase in NADPH during *Dictyostelium* development [42].

The increase in TKT activity might facilitate the need to recycle the excess of ribose-5-phosphate, a metabolite that could be accumulated as a result of the low requirement of nucleic acid synthesis as well as from RNA degradation during *Dictyostelium* development [43,44]. The activity of TKT would generate intermediates for glycolysis or glucose-6-phosphate that can enter again the oxidative phase to generate more NADPH. As autophagy is activated during development to support the recycling and the energy requirements of the cell, it is tempting to speculate that Atg1 might regulate this important pathway in concert with autophagy. Indeed, more work is needed to determine whether this regulation involves a direct phosphorylation of TKT by Atg1, which might alter the activity properties of the enzyme, or there is an indirect route linking autophagy to TKT activity.

## 4. Conclusion

We have investigated the network of protein–protein interactions by yeast two-hybrid analysis of the conserved autophagic proteins Atg1 and Atg8 in *D. discoideum*. These analyses confirmed expected interactions described in other organisms but also identified novel potential interactors. Gene disruption of the Atg1 interactors identified Atg13, Atg101 and two potentially new autophagy regulators (AreA and AreB). AreA, which shares some similarity with E3-ubiquitin ligase, interacts with Atg101 and functions as a negative regulator. Atg1 was also found to interact with the pentose phosphate pathway enzyme TKT. The activity of the endogenous enzyme is affected by changes in the expression levels of Atg1, which suggests a possible crosstalk between autophagy and the pentose phosphate pathway in *Dictyostelium* and human cells.

## 5. Material and methods

### 5.1. *Dictyostelium* cell growth, transformation and development

Wild-type Ax4 strain was used for genetic manipulation, knockout generation and overexpression of the different genes analysed in this study. *Dictyostelium discoideum* was grown in HL5 medium (Formedium) supplemented with 10% glucose and penicillin–streptomycin (10 000 U ml^−1^ penicillin and 10 000 µg ml^−1^ streptomycin; Gibco).

Starvation experiments were performed in PDF medium (20 mM KCl, 9 mM K_2_HPO_4_, 13 mM KH_2_PO_4_, 1 mM CaCl_2_, 1 mM MgSO_4_, pH 6.4).

Growth in association with bacteria was made on SM plates (10 g l^−1^ glucose (Sigma), 10 g l^−1^ peptone (Difco), 1 g l^−1^ yeast extract, 0.5 g l^−1^ MgSO_4_, 1.8 g l^−1^ KH_2_PO_4_, 0.6 g l^−1^ K_2_HPO_4_ and 20 g l^−1^ agar). A lawn of bacteria *Klebsiella planticola* was seeded before plating *Dictyostelium*. Cells were maintained in a standard microbiology incubator at 22°C.

Transformation was carried out by electroporation as described previously [45]. All genome information has been obtained from Dictybase [46,47].

### 5.2. Plasmid constructs

The extrachromosomal plasmid ptx-GFP was used to express Atg1 complex proteins for the analysis of their subcellular localization (Tom Egelhoff, GenBank accession number: AF269237). Plasmids with additional tags were made adding HA or FLAG sequences between the SacI and BamH1 sites of ptx-GFP, originating ptx-GFP-HA and ptx-GFP-FLAG. Two new alterations were introduced to ptx-GFP-HA and to ptx-GFP-FLAG that consisted in the removal of GFP from both plasmids, creating ptx-HA and ptx-FLAG. The plasmids pDM430 and pSJSK489 (Jason King, Beatson Institute for Cancer Research, Glasgow) expressing GFP-Atg8 and GFP-Atg18, respectively, were also used as autophagic markers [12]. Plasmid pA15/GFP-Apg1 expressing GFP-Atg1 was kindly supplied by DictyStock Center, Northwestern University in Chicago, IL, USA.

Autophagy-related genes were amplified by PCR in full-length with the exception of Atg1 and Atg13, which were divided in two separated fragments due to the high amount of TAA repeats present in these genes. Atg1 N-terminal expands from nucleotide 1 to 783 and Atg1 C-terminal from nucleotide 1172 to 2000. The Atg13 fragments were the Atg13 N-terminal (nucleotides 1 to 660) and Atg13 C-terminal (nucleotides 1218 to 2397).

For knockout constructs, a blasticidine resistance cassette was surrounded by two flanking regions of the target gene to allow gene disruption by homologous recombination. After PCR amplification, the flanking regions were purified and digested with the adequate restriction enzymes and cloned in a pBlueScript II vector containing the blasticidin resistance cassette described previously [48].

The mammalian plasmids pMXs-IP-EGFP-ULK1 and pMXs-IP-EGFP-ULK1(K46N) were a gift from Noboru Mizushima (Addgene plasmids nos. 38193 and 38197) [37]. The plasmid pMXs-IP-EGFP-TKT was generated by cloning human TKT by PCR in pMX-IP-EGFP plasmid using the EcoRI site.

### 5.3. Yeast two-hybrid

Yeast two-hybrid assays were carried out in *S. cerevisiae* TAT7 (*MAT*α* ade2–101 his3-Δ200 leu2-Δ1 trp1-Δ901gal4 gal80 LYS2::lexAop-HIS3 ura3::lexAop-lacZ*).

For yeast two-hybrid screening, plasmids encoding LexA fusion proteins were constructed by inserting a PCR fragment containing complete cDNAs of the genes indicated in the Results section into the BamHI or SalI sites of pBTM116 [49] with a substitution of TRP1 by URA3 marker (pLexA(U)).

Plasmids encoding these genes were co-transformed with a *D. discoideum* library ligated into pAD-GAL4-2.1 (Stratagene). For the pairwise analysis of interactions, the atg genes were also inserted in BamHI or SalI sites of pACT2 (Clontech) and pLexA(1–202)+PL [50].

The yeast two-hybrid library was constructed using mRNA isolated from growing cells and cells starved for 4 h. mRNA isolation was performed with the Absolutely mRNA TM Purification Kit (Stratagene) following manufacturer's instructions. The cDNA and the library were carried out according with the following kits from Stratagene: HybriZAP-2.1 XR Library Construction Kit and HybriZAP-2.1 XR cDNA Synthesis Kit. Yeast two-hybrid screen for interaction of Atg proteins was initiated with the pre-transformation of pLexA(U) derivatives followed by co-transformation of the cDNA library ligated into pAD-GAL4. His+ transformants were selected in the presence of 5 mM 3-aminotriazole and subsequently screened for β-galactosidase activity using a filter assay [51].

### 5.4. Confocal microscopy

*Dictyostelium discoideum* cells expressing fluorescent proteins (GFP and RFP) were plated in p100 plates and grown in low cell density for at least one week with a maximum of two weeks. For *in vivo* visualization, approximately 10 000 cells were transferred to an *in vivo* Ibidi µ-Slide eight-well slide and the fluorescence watched directly in a confocal microscope spectral LSM710 (Zeiss). The preferred objective for these experiments was the 63× Plan-APOCHROMAT.

### 5.5. Pull-down experiments and western blot

*Dictyostelium discoideum* cells (1 × 10^7^) co-expressing the indicated GFP- and the HA-tagged proteins were suspended in 200 µl of GFP-tap Lysis buffer (10 mM Tris/HCl pH 7.5; 150 mM NaCl; 0.5 mM EDTA; 0.5% NP-40) complemented with protease inhibitor cocktail (104 mM 4-(2-aminoethyl)-benzenesulfonyl fluoride, 80 µM aprotinin, 4 mM bestatin, 1.4 mM E-64, 2 mM leupeptin, 1.5 mM pepstatin A) for 30 min. The extract was centrifuged at 13 000 r.p.m. for 10 min and the pellet was discarded. The extract was diluted with 650 µl dilution buffer (10 mM Tris/ClH pH 7.5; 150 mM NaCl; 0.5 mM EDTA) with protease inhibitor cocktail and incubated overnight at 4°C in a rotated wheel with GFP-trap beads (Chromotek), previously washed with dilution buffer three times. The next day, the beads were washed five times with dilution buffer and suspended in 30 µl running buffer (125 mM Tris/HCl pH 6,8, 4% (p/v) SDS, 20% glycerol, 100 mM dithiothreitol (DTT) and 0.004% bromophenol blue). The sample was boiled at 100°C for 5 min and loaded into a SDS-PAGE gel for analysis. Analysis of cell lysates was performed by electrophoresis in SDS-PAGE gels and protein transfer to PVDF BioTrace membranes (Pall Corporation). Membranes were incubated with blocking solution (5% milk powder in TBS 1× with 0.05% Tween-20) at room temperature and, subsequently, with the appropriate primary antibodies prepared in blocking solution at 4°C overnight. Finally, the membranes were incubated with the secondary antibodies diluted in TBS-T (0.136 mM NaCl, 20 mM Tris Base, 0.05% Tween-20) for 1 h at room temperature and the antibody binding was visualized using the ECL detection system (GE Healthcare). Films were exposed at different times to ensure the bands were not saturated. Images were acquired with a SNAPSCAN e42 (Agfa).

### 5.6. Proteolytic cleavage assay for autophagy flux

The flux assay was performed as described previously [22,23]. The different *Dictyostelium* strains transformed with the construct GFP-PGKA were grown in HL5 with the appropriate antibiotics. The cells were harvested by centrifugation and washed with PDF once. After centrifugation, cells were resuspended in the appropriate volume of PDF (or HL5) and deposited in six-multiwell plates (10^6^ cells in 1 ml). NH_4_Cl was added to reach a concentration of 150 mM and incubated during 2 h at 22°C. After 2 h, the same amount of NH_4_Cl was added again and further incubated during 2 additional hours. After the treatment, cells were resuspended in the wells by pipetting, and transferred to Eppendorf tubes, pelleted by centrifugation and resuspended in 50 µl of RIPA buffer (50 mM Tris/HCl pH 8, 1% NP-40, 0.1% SDS, 0.5% sodium deoxycholate, 150 mM NaCl, 2 mM EDTA) supplemented with protease inhibitors (Sigma). The cells were kept in ice for 30 min to allow complete cell lysis. The amount of protein in the extract was determined by the Bradford method, and 10 µg of protein complemented with the loading buffer were boiled at 100°C during 5 min and the proteins separated in a standard 10% acrylamide SDS-PAGE gel.

### 5.7. Transketolase activity assay

*Dictyostelium discoideum* cells (1 × 10^7^ cells) in exponential growth or HEK 293T cells were scraped into a medium containing 20 mM Tris/HCl, 1 mM DTT, 1 mM EDTA, 0.2 mM phemylmethanesulfonyl fluoride (PMSF), 0.2 g l Triton X-100 and 0.2 g l sodium deoxycholate, at pH 7.5. The cell extracts were allowed to lyse on ice during 30 min (pipetting vigorously every 10 min) and centrifuged at 13 000 r.p.m. for 5 min at 4°C to eliminate portions of the cell that were not lysed.

The activity of the enzyme TKT was assayed by measuring spectrophotometrically the rate of NADH consumption at 340 nm from 0.15 mM NADH, 5 mM MgCl_2_, 0.1 mM thiamine-pyrophosphate, 2 mM ribose-5-phosphate and 1 mM xylulose-5-phosphate, in 100 mM Tris/HCl at pH 7.6, using glycerol-3-phosphate dehydrogenase and triose phosphate isomerase as coupling enzymes. G6PDH activity was determined by measuring the rate of NADPH production at 340 nm from 0.5 mM NADP^+^, 5 mM MgCl_2_ and 2 mM glucose-6-phosphate in 100 mM Tris/HCl at pH 7.6 [52].

## Supplementary Material

Suplementary_Figures_legend.docx

## Supplementary Material

Supp_Fig1

## Supplementary Material

Supp_Fig2

## Supplementary Material

Supp_Fig3

## Supplementary Material

Supp_Fig4

## Supplementary Material

Supp_Fig5
