# Supplementary material for: Dissecting the function of Atg1 complex in Dictyostelium autophagy reveals a connection with the pentose phosphate pathway enzyme transketolase
Source: Open Biol. 2015 Aug 5;5(8):150088. doi: 10.1098/rsob.150088 (PMC4554924; doi:10.1098/rsob.150088)
Supplement: Suplementary_Figures_legend.docx [file rsob150088supp1.docx]

**Suplementary Figures 1-5**

**Schematic representation of the gene disruption and analysis by PCR.** The black boxes represent the gene structure with the introns and the final location of the Blasticidine (BS) cassette after homologous recombination. Grey boxes correspond to the approximate location of the flanking regions that surround the BS cassette in the knock-out plasmids. PCR analysis was performed to verify homologous recombination in the isolated clones. Two pair of oligonucleotides were used, labeled as 1,2 and 3,4. The first two pair (1-2) surrounds the location of the BS cassette. The lack of amplification in the mutant indicates the presence of the BS cassette, which is too large for efficient amplification under these conditions. As a control for PCR performance two oligonucleotides from a different locus was used. The second pair of oligonucleotides were designed to amplify only in the DNA of the disrupted strain since one of the oligonucleotides is located in the BS cassette and the other one is located in a locus that is outside of the region covered by the flanking DNA. In this case a positive amplification indicates that the BS cassette is inserted in the correct locus. The expected results and examples of the different PCR amplifications are shown.
