# Supplementary figures and images for: Dissecting the function of Atg1 complex in Dictyostelium autophagy reveals a connection with the pentose phosphate pathway enzyme transketolase
Source: Open Biol. 2015 Aug 5;5(8):150088. doi: 10.1098/rsob.150088 (PMC4554924; doi:10.1098/rsob.150088)

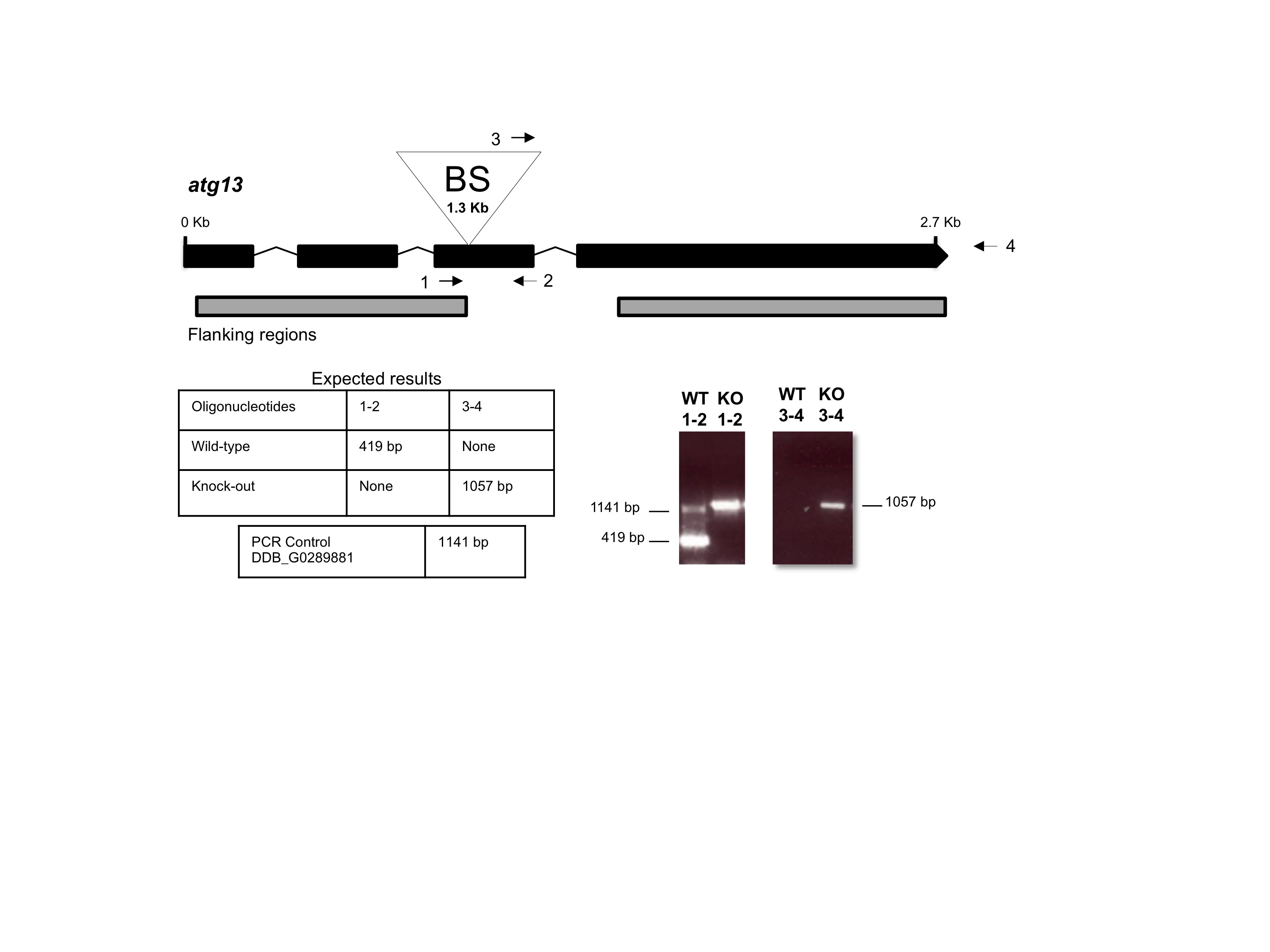

Supplement: Supp_Fig1 [file rsob150088supp2.jpg]

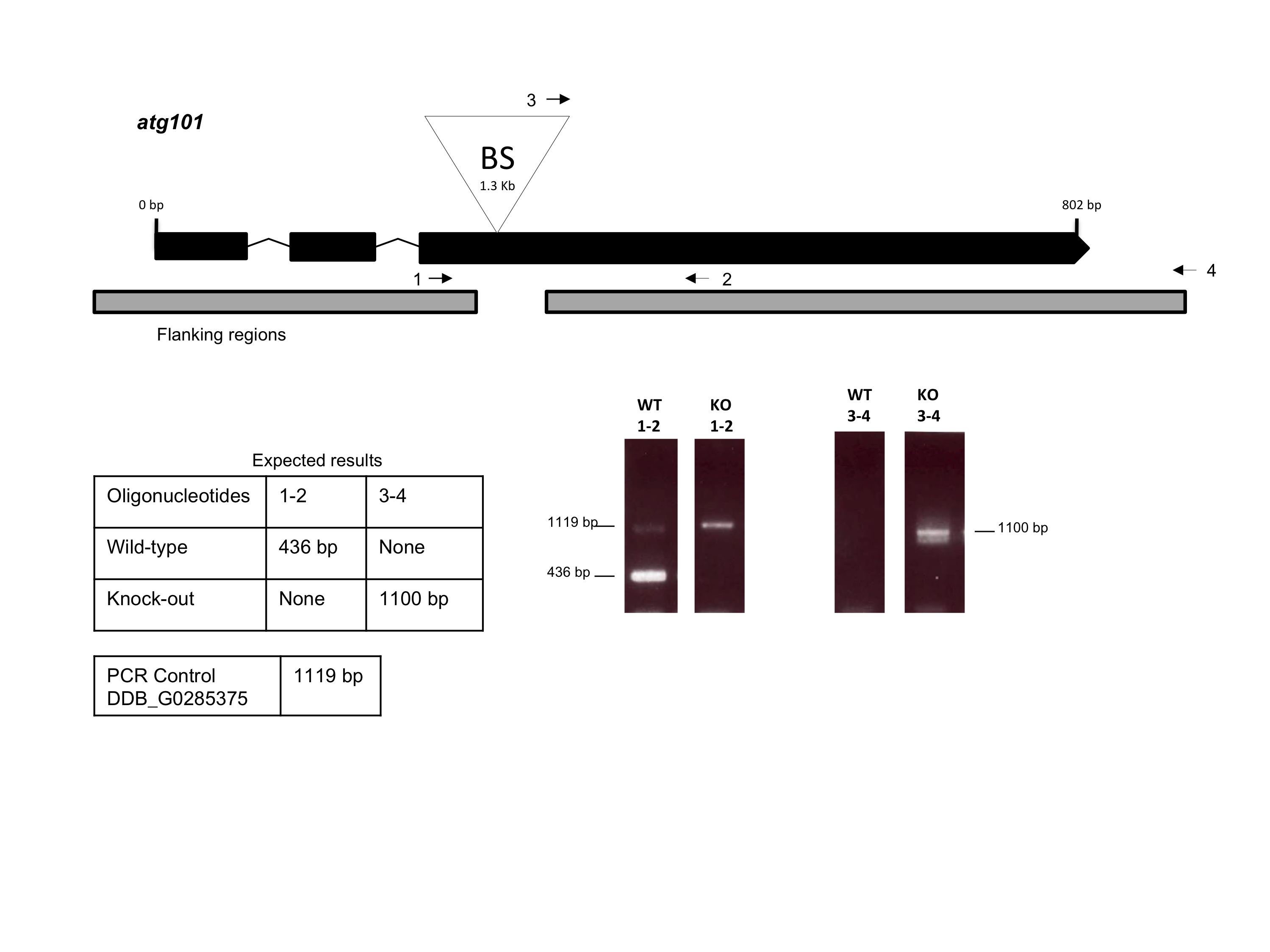

Supplement: Supp_Fig2 [file rsob150088supp3.jpg]

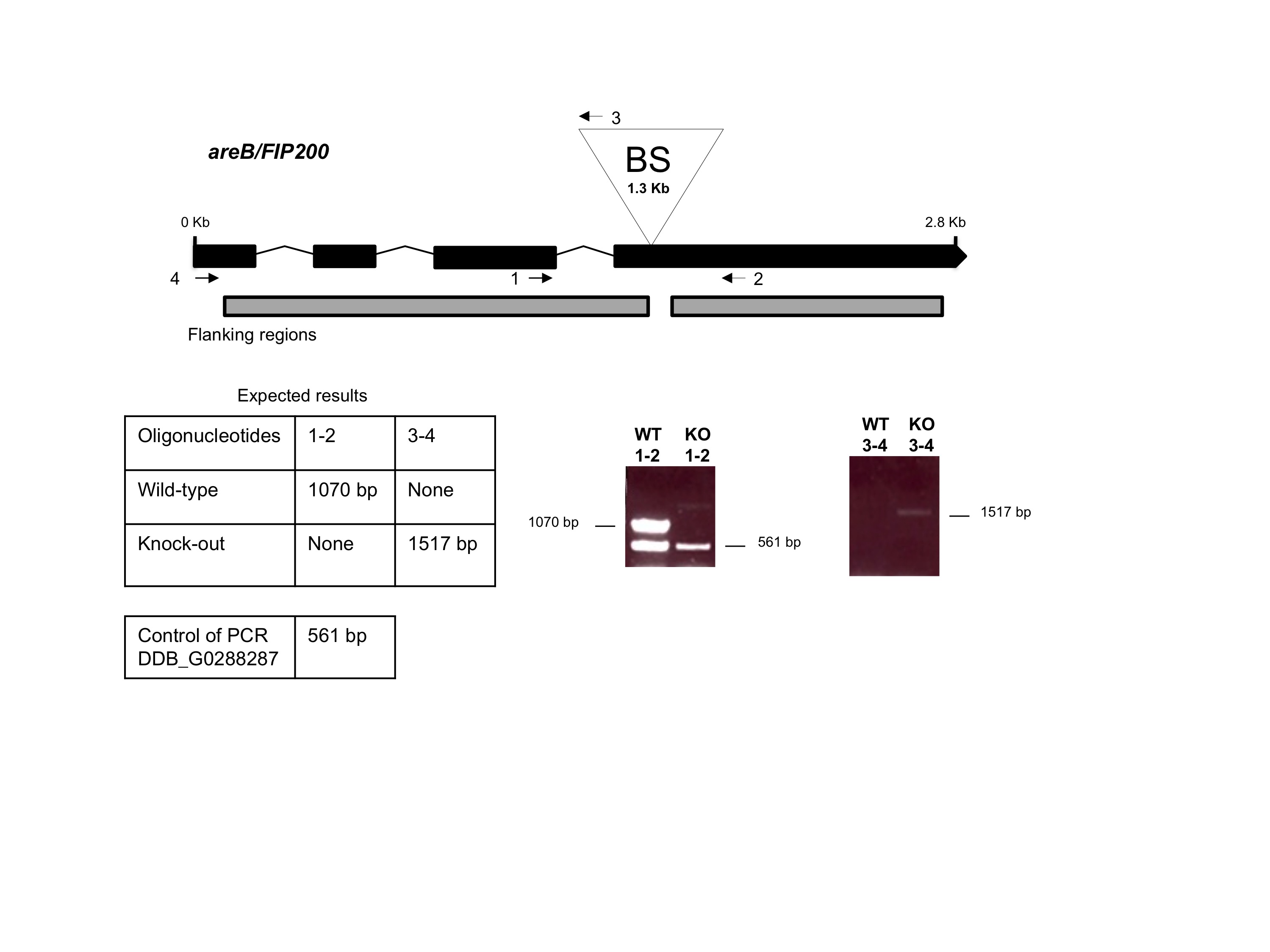

Supplement: Supp_Fig3 [file rsob150088supp4.jpg]

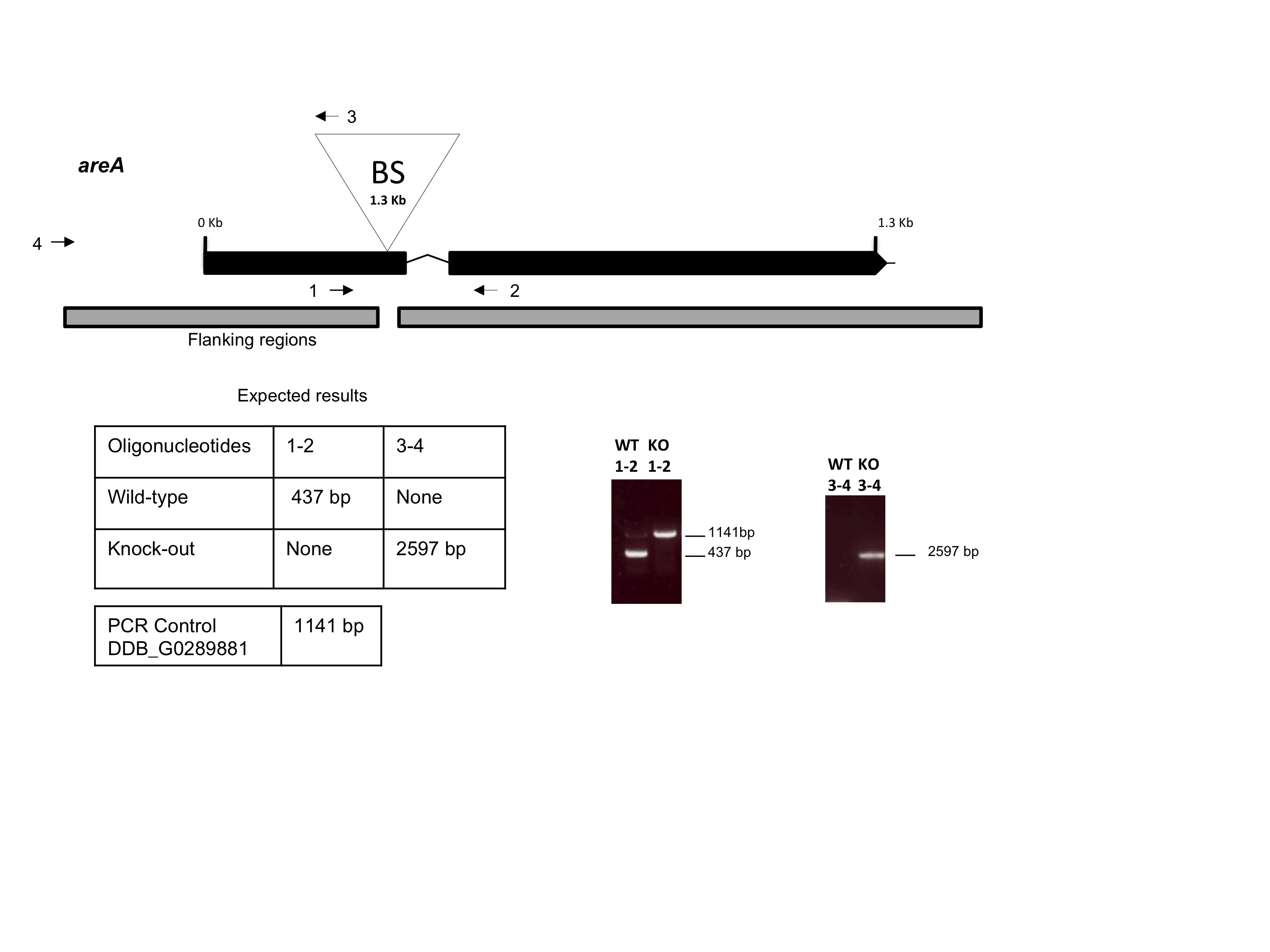

Supplement: Supp_Fig4 [file rsob150088supp5.jpg]

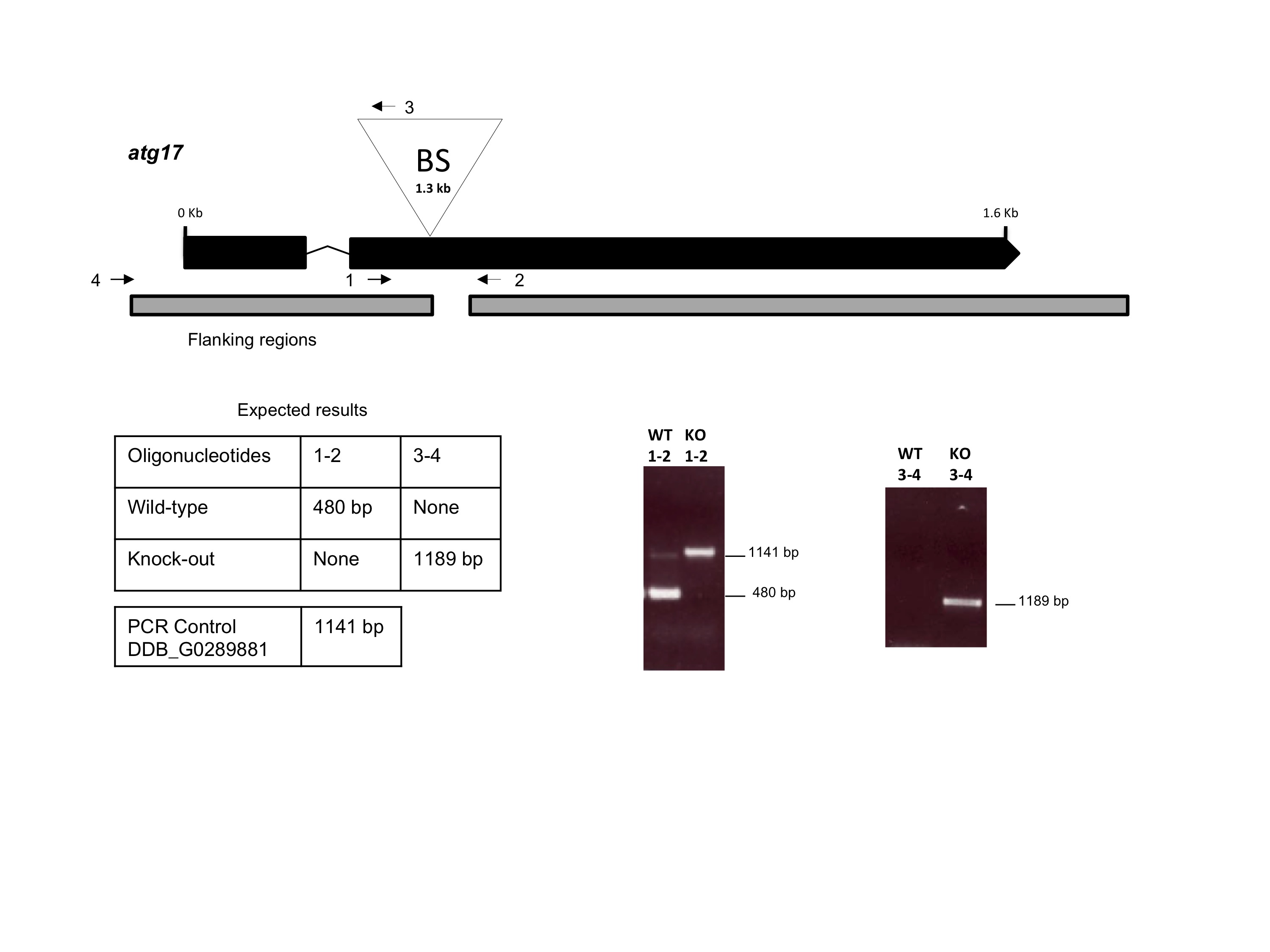

Supplement: Supp_Fig5 [file rsob150088supp6.jpg]
